# Exploring gaps in monitoring and evaluation of male engagement in family planning

**DOI:** 10.12688/gatesopenres.12927.1

**Published:** 2019-04-08

**Authors:** Bridgit M. Adamou, Brittany S. Iskarpatyoti, Chris B. Agala, Carolina Mejia

**Affiliations:** 1 MEASURE Evaluation, University of North Carolina at Chapel Hill, Chapel Hill, NC, 27516, USA

**Keywords:** Family Planning, Men, Male Engagment, Monitoring, Evaluation, Indicators

## Abstract

**Background: **Male engagement is becoming more common in family planning (FP) strategies and interventions, yet effective monitoring and evaluation (M&E) of this approach lags. This review sought to understand how male engagement in FP is defined, identify gaps in M&E of male engagement and make recommendations.

**Methods: **We conducted key informant interviews and a desk review of peer-reviewed articles and gray literature, including national FP strategies and policies.  We then facilitated an online forum with experts in the field of male engagement in FP to provide feedback on our proposed indicators for male engagement in FP to reach consensus on and validate key indicators.

**Results: **Although there is no universal definition of male engagement in FP, the most common definition is the inclusion of men in FP programming as FP clients, supportive partners, and agents of change. The most common approach was engaging men as clients exclusively, followed by engaging men as partners. Few papers reported on programs that engaged men across the full spectrum of the definition. There’s significant variation in the degree to which male engagement in FP is included in M&E, planning, and approaches. Few programs reported findings disaggregated by sex and by contraceptive method, making it difficult to determine the effect of programming on male use of methods. There is a dearth of indicators for measuring male engagement in FP in national strategies and policies. Other gaps are a lack of core indicators for male engagement, qualitative indicators, and indicator reference sheets for many commonly used indicators. Among over 100 indicators being used to monitor and evaluate male engagement in FP, 15 key indicators were identified and validated, with accompanying guidance.

**Conclusions: **As programming for male engagement in FP increases, coordinated efforts should be made to improve the systems that collect, analyze, and use data.

## Introduction

### Background

For more than two decades, gender equity has been widely recognized as a prerequisite for better health and has been integrated in global development goals. A prominent shift occurred at the 1994 International Conference on Population and Development, in Cairo, with the global call to action for a broader and more rights-based health agenda that included both women and men to address harmful gender norms and values, reproductive health (RH) for all, and shared responsibility for family planning (FP) ( United Nations Population Fund (UNFPA), 2014; United Nations Population Information Network, 1994). Following the International Conference on Population and Development, the Interagency Gender Working Group (IGWG) was established in 1997 by the U.S. Agency for International Development (USAID), USAID-funded cooperating agencies, and nongovernmental organizations (NGOs) with the goal of improving sexual and reproductive health (SRH) and HIV/AIDS outcomes, by promoting the integration of gender approaches in population, health, and nutrition programming ( Caro *et al.*, 2003). In 2000, the United Nations’ Millennium Development Goals set time-bound global development targets that included a specific gender equality and women’s empowerment goal (Goal 3) ( Kabeer, 2005; Sachs & McArthur, 2005). The succeeding Sustainable Development Goals, adopted in 2015, include a broad gender equality goal (Goal 5) that highlights the importance of SRH and reproductive rights ( Fredman *et al.*, 2016; Magar, 2015).

The focus on addressing gender inequalities to optimize health outcomes resounds in the field of FP. However, global FP initiatives, including Family Planning 2020, continue to concentrate primarily on women, with less attention paid to men ( Hardee *et al.*, 2016). Although some FP programs include men as an integral part of their intervention strategy, men are more commonly involved as gatekeepers or decision makers for women’s health or as “add-ons” in activities that focus on providing information and services to women ( Geleta *et al.*, 2015; Raj *et al.*, 2016).

Efforts to expand the vision of strategically engaging men in FP and RH have been slow but steady ( Dunn & Gage, 2010). Gender experts agree that men should be encouraged to be supportive partners of women’s RH while also meeting their own RH needs, and engaged as agents of change in families and communities ( Greene *et al.*, 2006). Constructive male engagement in FP entails a thoughtful, gender-sensitive approach that places gender equality and women’s empowerment on equal footing with other desired outcomes ( Gilles, 2015). Constructively engaging men, including adolescent boys, to be users of RH services themselves, shifting gender norms, and improving communication and joint decision making in couples can be challenging and require long-term efforts. Moreover, it is resource-intensive to demonstrate the impact of these efforts. In this report, the term “male engagement” is used synonymously with “constructive male engagement.”

Although male engagement is becoming more common in FP strategies and interventions, effective monitoring and evaluation (M&E) of this approach lags. Previous research on male engagement found the following M&E challenges: lack of clear behavioral objectives, limited data on men in RH and FP, lack of a common set of indicators on male engagement in FP, difficulty in capturing the complexity of gender, and complications in identifying or measuring gender outcomes ( Dunn & Gage, 2010). A gap remains in how to address these M&E challenges to move the field of male engagement in FP forward. Box 1 gives a plain English summary of this study.

Box 1. **Plain English summary**

Family planning programs are increasingly including men, however, the means of monitoring and evaluating how men are engaged is not well-developed. This review attempted to understand how male engagement in FP is defined, identify gaps in how male engagement in FP is monitored and evaluated, and make recommendations to address these gaps. We conducted key informant interviews and a literature review that included national FP strategies and policies. Following our review, we facilitated an online forum with experts in male engagement in FP to reach consensus on and validate key indicators. We found there is no universal definition of male engagement in FP. We determined that the most common definition is including men as FP clients, men who support their female partners’ FP choices, and men promoting positive male gender norms and FP. The most common approach was engaging men as clients exclusively, followed by engaging men as partners. The degree to which male engagement is included in how FP programs are planned, monitored, or evaluated varied significantly. Few programs reported findings by sex and by contraceptive method, making it difficult to determine the effect of programming on male use of FP methods. There aren’t enough indicators for measuring male engagement in FP in national strategies and policies. Key indicators for male engagement in FP were not identified and indicator reference sheets for many commonly-used indicators were missing. As programming for male engagement in FP increases, efforts should be coordinated to improve the systems that collect, analyze, and use data.

### Research objectives

The purpose of our research was to identify gaps in M&E of male engagement in FP, which we accomplished by implementing the following activities:

- Establishing a uniform framework for defining male engagement in FP programs, with clear behavioral objectives for each level of male engagement.
- Identifying existing indicators to track male engagement in FP, such as male FP service use, use of male FP methods, and other aspects of constructive male engagement (e.g., involving men as partners in FP decision making in behavior change communication activities and including men in efforts to address harmful gender norms).
- Identifying areas of male engagement for which there are measures and where appropriate measures are lacking.
- Analyzing existing indicators and systematically identifying strong indicators for M&E of male engagement.

We sought to review the landscape of M&E of male engagement in FP, identify gaps, and make recommendations to address the gaps in measuring male engagement across the male engagement framework. Our findings contribute to the goal of improving and applying methods, tools, and approaches to address RH information challenges and gaps.

## Methods

We expected information on male engagement in FP to vary based on the type of documentation reviewed (e.g., journal article versus program documentation). To understand how male engagement in FP is defined and measured, and the successes and challenges of M&E of such engagement, the study team conducted a desk review of peer-reviewed articles, gray literature, and national FP strategies and policies. We also conducted key informant interviews (KIIs) with staff from organizations that are currently implementing or have recently implemented activities involving male engagement in FP to obtain more in-depth knowledge about how these activities are monitored and evaluated, including successes and challenges. We collected indicators from both the desk review and KIIs.

### Desk review

We conducted a document review of published peer-reviewed and gray literature on male engagement in FP. Materials were identified through a literature search that included articles written in English and published between January 1996 and April 2016. The search was not bound by geographic location so that the widest possible range of sources could be captured. The gray literature included reports, working papers, research briefs, but not conference abstracts or posters, webinars, or presentations. Databases searched were PubMed, Scopus, Web of Science, Popline, USAID’s Development Experience Clearinghouse, and Google Scholar. The term “family planning” was searched in combination with “male/men’s engagement,” “male/men’s participation.” and “male/men’s involvement.”

The initial search yielded 293 publications. After eliminating those whose titles and abstracts did not meet our search criteria, 118 publications were extracted and entered in an Excel spreadsheet on a SharePoint website specifically created for this activity. Two members of the four-member study team reviewed the publications and excluded those that did not include: FP; an intervention; did not explicitly mention male involvement, engagement, or participation; or were redundant (i.e., another article covering the same intervention, study, or evaluation appeared in the database). The analysis resulted in a total of 72 relevant publications.

We abstracted the following information for each of the peer-reviewed and gray literature publications:

- Title, author, and publication year
- Organization
- Country and region
- How men are addressed (partners, clients, and/or agents of change)
- Intervention description
- Description of M&E methods identified (e.g., service statistics, focus group discussions, client-provider observations)
- Indicators or measures
- Type of indicators (qualitative/quantitative)

Using the Google search engine, we searched national FP or RH strategies to find mention of male engagement and, if it was found, how male engagement was being measured, if at all. “Strategy,” “policy,” and “framework” were included in the search terms, as was the term “RH,” because many countries include FP in their RH strategies. All USAID Population and Reproductive Health priority countries were searched individually, yielding 18 available FP/RH policies, representing 75 percent of the priority countries. We found an additional five strategies through a general (Google) search, bringing the total to 23 national FP/RH policies, strategies, and frameworks. Only policies produced in the past decade (2006 to 2016) were included. When the search yielded multiple FP strategies for a country, we included only the most recently approved strategy. However, at the time of writing, six strategies had expired, based on the time frame covered by the strategy.

We created an Excel spreadsheet to collect the following information from the national FP strategies/policies:

- Country
- Name of document and year, or years the policy or strategy covers
- How male engagement in FP is addressed
- Indicators pertaining to male engagement in FP

An Excel spreadsheet with the 72 publications from the literature review and the 23 national FP/RH policies, strategies, and frameworks can be found on Figshare ( Adamou, 2019).

### KIIs

We conducted KIIs to compare with the information we obtained from the literature review, and to gather in-depth information on male engagement indicators and M&E challenges.

We used the snowball sampling strategy to recruit interview participants. First, we developed a list of nine organizations to contact from the desk review of programs and organizations, based on whether they had published on male engagement in FP in the past decade. We identified key informants from the publication authors. Additional names were obtained by drawing from our professional connections, and in-person contacts at the May 2016 Women Deliver conference. Next, we contacted 14 key informants by email to explain the activity and schedule a time for the KII. All the key informants were from organizations that implement FP programs; most were based in the United States. The individuals had backgrounds in M&E, implementing male engagement in FP interventions, or both.

One person did not reply. Two contacts referred us to a colleague (who was already listed as one of our original 14 contacts) whom they felt would be better suited to provide the needed information. Two others showed interest in being interviewed but did not respond to our emails for setting up a time for an interview. Using an interview guide, we interviewed a total of nine people from eight different organizations. (Two individuals from the same organization were interviewed at the same time.) Interviews were conducted by phone or Skype. The interviews lasted between 30 and 60 minutes. After conducting the interviews, we reached a point of information redundancy. The interview guide, along with deidentified transcripts of the interviews can be found on Figshare ( Adamou, 2019).

The KIIs covered three areas:

- Information on how the organization or project defines male engagement in FP
- How the organization or project monitors male engagement in FP activities, including what indicators are used
- How the organization or project evaluates male engagement in FP programs, including what indicators are used and the challenges, best practices, or lessons learned

### Online forum

To validate the high-quality indicators for male engagement in FP that were selected through this review, we conducted a four-week online forum with experts in male engagement in FP to gather feedback. We asked forum participants to share their experiences with and reactions to the proposed indicators, discuss potential missing key indicators, and share solutions to reach consensus on key indicators for measuring male engagement in FP. To introduce the forum, we conducted a kick-off webinar, “Selecting Key Indicators for Male Engagement in Family Planning.” Both the webinar and online forum were led by the lead authors of this review. Leveraging the Male Engagement Task Force, we invited experts in the field of male engagement in FP to participate in the webinar and forum. The webinar provided an overview of MEASURE Evaluation’s review of the gaps in M&E of male engagement in FP; explained the criteria that were applied to select strong indicators; and introduced participants to the online forum for gathering feedback on the indicators and informing the selection of key indicators for male engagement in FP ( Iskarpatyoti & Adamou, 2018).

Forty-two people joined the online forum, hosted by Google Groups. In addition to members of the Male Engagement Task Force, we encouraged participants to forward the forum invitation to organizational or project staff who had experience in male engagement in FP and/or M&E. Participating experts came from a variety of backgrounds, organizations, and countries. Through the online forum, we facilitated a discussion over a period of four weeks, organized as follows:

- Week 1: Launch forum and introduce ourselves
- Week 2: Discuss the indicators for men as clients
- Week 3: Discuss the indicators for men as partners
- Week 4: Discuss the indicators for men as agents of change; summarize the discussion and close the forum

### Ethics approval and consent to participate

The study team applied to the University of North Carolina at Chapel Hill’s Office of Human Research and Ethics for approval to conduct the KIIs. The Office of Human Research and Ethics determined that this study did not constitute human subjects research as defined under federal regulations, and therefore, did not require institutional review board approval. Participants were informed of the purpose of the KII, including an overview of the topics to be covered, how the data would be used, and how names or organizations would be referenced in the report. Verbal consent was obtained before each interview.

### Data analysis

We conducted a thematic analysis of the KIIs, reviewing how the organization/project monitors and evaluates its male-engagement-in-FP activities/programs, with a focus on the indicators used and the data sources. We entered the indicators provided from the KIIs in a master Excel spreadsheet, which also contained all the indicators related to male engagement extracted from the literature review.

The indicators that were discussed in the online forum were compiled in a table, along with all corresponding comments from both the participants and the facilitators.

### Identifying and collating indicators for male engagement in FP

Based on the desk review and KIIs, we compiled 103 output, outcome, and impact indicators currently used for measuring male engagement in FP and RH. We organized the indicators in a three-dimensional matrix according to the male engagement framework (i.e., men as clients, men as partners, and men as agents of change), the level of intervention (i.e., individual, community/facility, structural) and type of indicator (input, output, outcome, or impact). The individual level relates to men’s personal knowledge, attitudes, and practices. The community/facility level pertains to data collected at the health facility and/or community level, or to data that apply to health providers specifically. Indicators for the structural category measure changes at the larger, systemic level, such as guidelines, policies, laws, and the media. For the purposes of simplification, we list each indicator once. However, we recognize that some indicators may fit into multiple categories of interventions or approaches.

Although several input and process indicators were similar across projects, many were closely tied to specific program activities. These indicators were not included because they were designed for a specific project or NGO and were therefore too varied for the scope of this report. For monitoring purposes, we included a select number of output indicators that are common in male engagement programs but focused mainly on outcome indictors. For evaluation purposes, we included impact indicators. Myriad indicators on SRH and FP programs and services in general are described elsewhere (for example, MEASURE Evaluation’s FP/RH Indicators Database); however, they were not relevant enough for this research. We included general SRH indicators only if they directly affect or are affected by men’s involvement.

The indicators were copied verbatim from the desk review and indicator documents provided by the KIIs. Some indicators are broadly applicable, whereas others can pertain to a specific intervention. Although we acknowledge that the format and wording of the indicators vary, it was important to present them in their original form, because this provides a snapshot of the breadth and quality of the indicators that are being used to measure male engagement in FP.

Following the compilation of the indicators in an Excel spreadsheet, the study team systematically analyzed each one based on eight standard criteria, and scored each indicator based on a scale ( Table 1). For the binary scales, only indicators that met the criteria were assigned a point. For the criteria with a scale of one to three, indicators were assigned one point if the indicator did not meet the criteria, two points if the indicator somewhat met the criteria, and three points if the indicator met the criteria. Because of the subjectivity of the scales, three reviewers in the study team scored each indicator based on the eight criteria and an average score was calculated.

**Table 1.** Indicator criteria, definitions and scales.

Criteria	Explanation	
Specific	The indicator is specific to the change being measured. It is precisely formulated, not vague.	0, 1
Measurable	The indicator is easily monitored, and amenable to independent validation.	0,1
Attainable	The indicator requires data and information that can be collected.	0,1
Relevant	The indicator is appropriate to the subject of male engagement in FP and evaluation.	0,1
Commonly used	The indicator is frequently used by programs to monitor or evaluate male engagement in FP.	1, 2, 3
Validated and/or already collected in routine data collection	The indicator is already validated and/or used in routine data collection, such as DHIS 2, Demographic and Health Surveys (DHS), or other validated surveys.	0, 1
Generalizable	The indicator can be used across multiple types of FP interventions and is not specific to a method or process.	1, 2, 3
Applicable to FP programs sponsored by a variety of funding agencies, governments, or NGOs worldwide	The indicator can be used by any program/project regardless of implementing or funding agency.	1, 2, 3

FP, family planning.

Following our individual reviews, the study team met to analyze and compare the indicator rankings based on the average scores. Looking particularly closely at the indicators that received higher scores, the team analyzed and discussed each indicator with the indicator criteria in mind.

Based on this analysis, we identified 18 that would be considered strong, high-quality indicators for male engagement in FP. These indicators were put forth to the group of male engagement in FP experts in the online forum. After analyzing the input on the indicators via the online discussion, 15 indicators were selected as key. We collected available indicator reference sheets for these indicators. For indicators without indicator reference sheets, we adapted similar available sheets (e.g., we referenced the indicator reference sheets for female sterilization for the vasectomy indicators, none of which had existing indicator reference sheets).

## Results

### Defining and operationalizing male engagement in FP

We began this investigation by asking how male engagement in FP is defined and operationalized by projects, organizations, and countries, by conducting a desk review of peer-reviewed journals, gray literature, and country documents. Early program publications (primarily from the 1990s) varied widely in how male engagement was defined and approached, showing preference for engaging men as partners and gatekeepers to women’s health, or as clients by providing vasectomy services. More recent publications reveal increased uniformity in specifying what male engagement in FP entails, with the most commonly mentioned approaches to male engagement in FP aligning with a framework ( Table 2) that depicts men’s roles in three overlapping areas ( Greene *et al.*, 2006):

- Men as clients and beneficiaries: Those receiving FP methods or counseling on male-controlled and cooperative methods; addresses men’s FP needs
- Men as supportive partners: Those actively engaging as a full partner in FP issues, and communicating and negotiating fertility desires and FP use; engages men as supportive partners
- Men as agents of change: Those acting as leaders in shifting underlying community and cultural norms, attitudes, and behaviors toward women and girls and their place in families, communities, and societies at large; promotes gender equality as a means of improving men’s and women’s RH as an end in itself

**Table 2.** Programmatic areas, descriptions, and objectives in the Constructive Male Engagement Framework.

Area	Description	Programmatic objectives
**Men as clients**	Address men’s FP needs	Increase knowledge of healthy timing and spacing of births, modern contraceptives, and FP options for men. Promote increased demand, accessibility, acceptability, and use of male-controlled FP options, such as condoms and vasectomy, as well as Standard Days Method, which requires men’s active participation as a cooperative method. Ensure quality in provision of FP services to men.
**Men as** **partners**	Engage men as supportive partners	Improve healthy communication and joint decision making within couples. Expand men’s knowledge of and participation in their partner’s contraceptive planning and use (e.g., knowledge of partner’s method, fertility, and desired family size). Increase shared responsibility for decisions around contraception and protection against sexually transmitted infections and HIV. Promote men’s supportive and enabling role before and during pregnancy and childbirth, and responsibility as parents and caregivers in the family.
**Men as agents** **of change**	Promote gender equality as a means of improving men’s and women’s RH as an end in itself	Promote gender equitable fatherhood. Support advocacy against discriminatory SRH laws and policies. Encourage reflection on and challenge attitudes about gender roles to help shift assumptions and values that drive gender inequality.

Source: Adapted from Margaret Greene’s *Male Engagement in Family Planning Framework* (2006). FP, family planning.

Although there is no universal definition of male engagement in FP, we found consensus, adoption, and use of this framework by multiple international organizations, bilateral agencies, and the IGWG ( Doggett & Herstad, 2008; IGWG, 2016; International Planned Parenthood Federation, 2014; Population Reference Bureau, 2016; United Nations Population Fund & International Council on Management of Population Programmes, 2011). From this, we determined that the most common definition of male engagement in FP is the inclusion of men in FP programming as clients of FP services, as supportive partners, and as agents of change in the family and community. Whereas male engagement generally pertains to men, it also pertains to male youth.

Program reports and the KIIs revealed that each of the male engagement and FP approaches described was tied to specific, common programmatic objectives, as outlined in Table 2.

**Figure 1.**
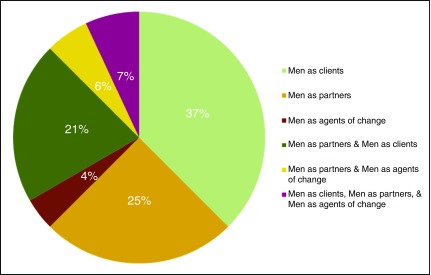
Approaches to engaging men in family planning programming.

Depending on the intervention or strategy, the inclusion of men in achieving the desired FP outcomes fell within one or more of the three categories. Of the 72 papers in the desk review (some of which were mentioned during the KIIs), the most common approach was engaging men as clients exclusively (n=27), followed by engaging men as partners (n=18) ( Figure 1). Few papers reported on programs that engaged men only as agents of change (n=3). One-third of the papers reported overlapping approaches, with 19 papers reporting on engaging men across two categories, and five papers reporting on engaging men across the full spectrum: as clients, partners, and agents of change.

The degree to which male engagement in FP was mentioned and included as a strategic approach in national FP/RH strategies, policies, and frameworks varied significantly. For example, Ethiopia’s *National Guideline for Family Planning Services* (2011) explicitly states that “males shall be addressed in family planning programs and services as users, promoters and decision-makers” ( Ministry of Health, Federal Democratic Republic of Ethiopia, 2011). The document lists several guidelines for how to achieve male inclusion, such as making FP services male-friendly, including men in the design and implementation of FP and RH services, and encouraging men to accompany their partners to FP visits. The Philippines, Zambia, and Mauritius also include several strategies in their FP/RH policies on how to involve men ( Department of Health, Philippines, 2006; Ministry of Health and Quality of Life, Republic of Mauritius, 2008; and Ministry of Health, Zambia, 2006). Other documents, such as Rwanda’s *National Family Planning Policy* (2012), mention promoting greater male participation in FP programs as one of the goals but make no further reference to men and do not include a strategy for how this goal will be achieved ( Ministry of Health, Republic of Rwanda, 2012). Four others, including Haiti’s *National Strategic Plan for Reproductive Health and Family Planning* (2013), do not contain a goal or strategy to engage men in FP or even mention male engagement ( Ministry of Health, Haiti, 2013).

Although we looked for trends by different variables that might affect how countries address male engagement in FP, such as region, predominant religion, USAID FP priority country, and global FP partnerships, among the 23 national FP/RH strategies reviewed, we found no clear trend for including male engagement in FP as a goal and/or programmatic approach among the countries. For example, countries in Africa were no more or less likely to include men in their strategies than were countries in Asia. The same could be said of predominantly Islamic countries. Mali, for example, listed specific activities for engaging men in FP ( Ministry of Health and Public Health, Mali, 2014) whereas Yemen did not include any strategy, activity, nor indicator related to male engagement in FP ( Ministry of Public Health and Population, Yemen, 2011).

### Measuring male engagement in FP

A challenge identified by one key informant, and confirmed in the literature review, is the ambiguity of some policymakers, program designers, and service providers around deciding whether men should be engaged in FP in the first place. This is because increased involvement of men in SRH may interfere with women’s ability to make FP decisions on their own and undermine women’s empowerment efforts. Because some countries have not yet made the decision to engage men in FP, there is no need for them to track male engagement in FP services and programs, as users, supportive partners, or agents of change.

Most of the key informants mentioned that the M&E of interventions that engage men in FP have lagged support for these programmatic approaches. As to the measurement of males as clients, this review found that few programs, particularly those that relied on routine national data, reported findings disaggregated by sex and by contraceptive method, making it difficult to determine the effect of programming on male use of methods.

Key informants discussed the difficulty of working within routine systems because the data collection tools that are available at health centers, such as patient registers and files, do not facilitate the tracking of progress in male engagement in FP. This is particularly true in contexts where male engagement in FP is not prioritized in national FP/RH strategies and where it is not tracked by routine health information systems. As stated by one informant, “In general, the information [on male engagement in FP] is hard to get. We use a lot of government forms, but there’s no place to capture the information.” Key informants mentioned that―except for monitoring condom use and vasectomies―other aspects of male engagement in FP, such as men as partners and men as agents of change, are more difficult to track because of such challenges as expense, time, and locating enough men to survey.

Among the country strategies and policies reviewed, although nearly all included indicators, few included indicators for measuring male engagement in FP. Approximately one-half of the strategies we reviewed do not have any indicators specific to male engagement in FP. Among those that do, most of the indicators would be specific to men only if disaggregated by sex: for example, FP counseling provided; percentage of the population with a favorable attitude towards an FP product, practice, or service; and percentage of eligible couples who access birth spacing services. A limited number of strategies include indicators specifically focused on men: for example, number of men attending SRH services; male sterilization coverage rate; and number of male participants reached by FP sensitization workshops. As stated by one key informant, “We need to come up with more cost-effective and easier ways to collect indicators. Otherwise, people aren’t going to collect information on them. The gap really shows in male engagement.”

Because of the lack of routine data on men in FP collected through larger health information systems, our key informants discussed the need to rely on program-specific M&E or the Demographic and Health Surveys (DHS). Program-specific M&E is generally tailored to the needs of the implementing organization and is therefore not consistent across projects and interventions. Programs implemented as randomized control trials, for example, have highly monitored implementation, collect large amounts of data, and are difficult to reproduce and sustain beyond the initial implementation. Programs may collect monitoring data through monthly reports, supervisory forms, or internal audits; however, these mechanisms are not routine and cannot be built into a systematic health information system.

Even in situations where the preferred health management information system (e.g., DHIS 2) is used to collect and aggregate data at the global, country, and local levels across several countries, the data may be limited to organizational or program use. For example, one key informant stated that they used DHIS 2 to track the number of FP users from service delivery sites and sales of condoms. Yet the organization’s proprietary rights over their data limit the data’s usefulness to other program implementers and researchers working in FP. These limitations often prevent triangulation and comparisons of data across interventions.

Although core FP indicators were developed and standardized more than 20 years ago, few commonly used indicators specifically capture male engagement in FP. Some RH indicators depend on sex and age disaggregation (e.g., service use; counseling); however, the data may not be collected or analyzed by sex/age in practice. Gender-sensitive measures may provide an opportunity to collect more nuanced information on male engagement, such as power relations in the household that may drive FP decision making; men and women’s perceptions of FP; and/or cultural norms around fertility. Promundo, an international NGO that focuses on engaging men and boys for gender equality, has spent almost two decades developing, testing, and validating its Gender Equitable Men (GEM) scale ( Pulerwitz & Barker, 2008), which many projects use to measure gender attitudes among men and women. However, based on our desk review and the KIIs, we found that such measures have not been integrated in any routine data collection tool, and therefore require organizations to dedicate additional resources to such data collection.

Relying on DHS to assess changes in gender outcomes among both men and women is restrictive. DHS data are collected on gender norms, but assessing FP outcomes is more challenging as questions on whether men have accessed FP services are not included in the main questionnaire. For example, questions on FP decision making are generally only asked of women; therefore, information on male engagement or joint decision making is indirect or partial. There is a male questionnaire in the DHS; however, not all country DHS include it for reasons that include time and cost.

Moreover, even when data are collected on men from other data sources, such as service statistics, they are rarely disaggregated by age, as is the norm for data on women. The key informants stated that, like FP data on women, data must be triangulated to fully understand the status of men’s involvement in FP, which in most cases implies data collection from different sources and by various methods.

Qualitative data, gathered from such methods as KIIs, program participant focus groups, observations, and case studies, are often required to complement quantitative measurements, especially because few indicators for male engagement in FP are collected through routine data collection. The desk review and KIIs revealed that qualitative data are essential to understanding the context in which programs are, or are not, successful and are helpful to understand the perceptions and attitudes that may drive male behavior.

## Discussion

Through our document review and KIIs, we found consensus on how male engagement in FP is defined. Among the three intersecting areas of male engagement, most FP programs or strategies that make a conscious effort to involve men focus primarily on men as clients. Programmatically, this is considered an easy target, because it is typically easier to design, monitor, and evaluate programs that increase men’s use of FP methods than programs that increase men’s participation in their partners’ contraceptive planning and use, or programs that improve gender equity. Few programs address men across the spectrum. This is partly because of the traditional focus on women in FP programs and activities, with men’s involvement being an ancillary strategy to improve women’s access to and use of FP, rather than approaching men as pivotal influencers of contraceptive use and fertility trends. In other words, programs that addressed men across the spectrum of male engagement in FP acknowledged men as key players in improving FP and gender equity outcomes.

By supporting *men as clients*, programs provide an opportunity for men to improve their ability to make informed choices about their fertility through male-centered FP education; awareness; and services, such as condoms, vasectomy, and couple-centered services. However, it is important that these programs not be gender-exploitative *, galvanizing men’s dominant position in certain cultural settings, by focusing on their needs and their control of FP rather than on the couple as a unit and the underlying gender relations.

Approaches that address *men as partners* reflect the idea that men and women should work as allies in efforts to improve the healthy timing and spacing of pregnancies, contraceptive prevalence rates, and other dimensions of FP. Many of these programs address men within the context of the couple, and encourage men to support and communicate openly with their partners and share in the decision making. However, these programs typically do not evaluate whether they are gender-exploitative or gender-accommodating *, by either intentionally or unintentionally maintaining men as gatekeepers or primary healthcare decision makers in the family, or whether they are pushing men and women as equal allies in sharing FP responsibility and action.

By emphasizing *men as agents of change*, program implementers examine the relationships between women and men in a gender-transformative * approach to support broader social change. These programs address the underlying cultural gender norms and expectations that drive FP attitudes and service use. They do not necessarily focus on specific FP services and to whom they are delivered; they often impact outcomes beyond FP alone, because the approach uses men’s social capital and leadership opportunities in the public sphere to advocate for women’s rights and access to contraceptive services and products at the policy level. Although these programs address structural norms that drive FP outcomes, their scope lands outside the aim of FP-specific programs. Nevertheless, the changes in gender norms and attitudes should still be monitored and evaluated in the context of male engagement in FP, because of the significant influence they have on fertility intentions, reproductive choice, and contraceptive use.

### Gaps in M&E of male engagement in FP

Because of the lack of attention to male engagement in several country FP/RH strategies, there is a lack of indicators for monitoring and evaluating male engagement. Given the importance of engaging men as FP users, influencers of FP use by their partners, and advocates for improvements in gender equality in society for improved FP outcomes, it is important that national FP/RH policies and strategies acknowledge men’s participation and include strategies for how men will be engaged. Including relevant indicators in policy-level documents will help encourage, guide, and track male engagement in FP interventions.

Among the indicators we found through our desk review and KIIs, not only do the sources of these indicators vary tremendously, so does their quality. For example, one of the more poorly worded indicators, which lacks both specificity and clarity, is “Perception of providers to men in FP.” Likewise, “Greater resources available for gender equality and male involvement in FP campaigns” would be improved by making the indicator nondirectional and more specific. Thus, although plenty of indicators are being used to track male engagement in FP globally, another M&E gap we discovered was lack of identification of *high-quality* indicators. This was particularly true in the area of men as agents of change.

A set of core indicators for male engagement has not been identified. Additionally, the indicator reference sheets for many commonly used indicators are incomplete (e.g., missing definitions of key terms and guidance on how to accurately capture the information or calculate the indicator or the data sources) or nonexistent. For example, none of the indicators related to vasectomies had indicator reference sheets, implying that the data are not being captured consistently or completely.

By examining more than 100 indicators that are being or have been used to measure male engagement in FP, we found evidence of the breadth of male engagement in FP activities being implemented. Although this level of involvement is encouraging, the tracking of these activities tends to be resource-intensive, because most of the indicators (except for condom distribution and vasectomies performed) have not been integrated in any routine data collection tool. This creates a gap: the field of FP advances―in terms of acknowledging and capitalizing on men’s roles in contraceptive use and being agents of change in improving the health of families and communities―but it lacks standard M&E resources to track the engagement in a way that is both accurate and cost-effective.

Last, because indicators are typically quantitative, we did not find many qualitative indicators. That is not to say that qualitative measures for male engagement in FP are not collected and reported; they are not collected and reported in a way that allows the information to be reported against an indicator. We included a qualitative indicator in our recommended list of strong indicators to measure health providers’ perceptions of men accompanying their wives or partners to an FP visit. This is a common indicator among FP programs working to engage men at the facility level. Though it has not been validated, this indicator is attainable and relevant to the subject of FP and evaluation. It is often used across many types of FP interventions by different projects, regardless of implementing or funding agency. Nonetheless, the use of qualitative measures as indicators is an ongoing discussion in the field of M&E.

### Limitations

This review has limitations worth noting. First, our study may not represent all organizations conducting M&E of male engagement. Our initial intention was to interview three more people from partners implementing FP programs, but once we reached data redundancy, we decided not to pursue additional interviews. Secondly, we acknowledge that there are likely more indicators on male engagement in FP than the 103 we compiled. Although our list is not exhaustive, we are confident that it presents the most commonly-used male engagement in FP indicators, and that any others are just slight variations on the ones we have listed.

## Conclusion and recommendations

As programming for male engagement in FP increases, coordinated efforts should be made to improve the systems that collect, analyze, and use data for decision making. This review makes several recommendations to improve the M&E of male engagement in FP programs. The recommendations focus on using a standard definition of male engagement in FP; including male engagement in national FP/RH strategies; identifying and adopting key indicators; and employing existing data collection approaches and methods. The recommendations can form the basis for a guide on M&E of male engagement in FP programs to standardize the way male engagement in FP is conceptualized and measured.

### 1. Use a shared definition of male engagement in FP

The design of most national FP programs often excludes men, creating a gap in programming to address men’s needs in FP, planning for fatherhood, preventing unwanted pregnancies, and partners’ joint decision making in FP choices. This gap exacerbates gender inequality. Program designers, implementers, and evaluators should use a shared definition of male engagement in FP based on the three overlapping spheres of the male engagement framework: addressing men as FP clients, as partners, and as agents of change. By doing so, more effective strategies can be developed to address gaps in FP programming; therefore, they can improve gender equality both directly and indirectly. A shared definition will also help with the measurement of comparable programs and thus yield more comparable data.

Although many programs focus on one or two of the three approaches to male engagement in FP, understanding and addressing the full spectrum of male engagement will provide longer-term, more sustainable impact.

### 2. Include male engagement in national FP and RH strategies

Male engagement in FP and its measurement are not reflected as priorities in most national FP/RH strategies. This is a missed opportunity for countries, because there is overwhelming evidence of the importance and effectiveness of including men in FP/RH interventions and encouraging their participation. Without the national-level mandate and guidance on how to constructively engage men in FP, and how to effectively include them in FP interventions, monitoring and evaluating their contribution will continue to be challenging.

All the national FP/RH strategies we reviewed were developed with donor support, in consultation with international implementing partners. Donors and implementing partners therefore share the responsibility of advocating for the inclusion of men in national FP strategies and policies, and presenting the evidence for why this will be beneficial. Policymakers in ministries of health should consider evidence-based practices for achieving FP goals and objectives, and formally recognize the importance of male engagement in FP.

### 3. Use strong, high-quality indicators

Monitoring and evaluating FP programs that engage men is vital to determining the relative success of different strategies, providing data for program improvement, and presenting evidence of the impact of involving men. Evidence of impact entails health outcomes for men and women as well as changes in gender norms and dynamics.

The quality of indicators on male engagement in FP varies significantly, with many not meeting the conventional standards of good indicator design (i.e., the indicator is valid, reliable, precise, measurable, timely, and programmatically important) ( Frankel & Gage, 2016). There is also a significant knowledge gap as to which standardized indicators should be used to address all aspects of male engagement in FP—with the goal of increasing men’s use of FP, improving men’s role as supportive partners in decisions around FP, and encouraging men to be advocates for gender equality and improved FP access and services. Based on the indicators in use for this topic, we originally identified 18 strong, high-quality indicators for male engagement in FP that could be adopted by designers of male engagement in FP programs and initiatives. With the feedback gathered from the online forum, we settled on 15 key indicators ( Table 3). These indicators cover the full spectrum of male engagement in FP, including both programmatic focus (i.e., men as clients, men as partners, and men as agents of change) and the level of intervention (i.e., individual, community/facility, and structural). Many of the selected indicators pertain to use of condoms and vasectomy services, which are key desired behaviors for male engagement in FP.

**Table 3.** Recommended key indicators for monitoring and evaluation of male engagement in family planning (FP).

	Men as clients	Men as partners	Men as agents of change
Individual	• Percent distribution of contraceptive methods currently used by men or their sexual partners (outcome) • Percent of men who have ever used any male FP method or FP method that requires male cooperation (outcome) • Men’s condom use at last sex (outcome)	• Percent of men who support the use of modern contraception for themselves or their partners (outcome) • Percent of men who share in the decision making of RH issues with their spouse or sexual partner (outcome) • Percent of men who disagree that contraception is a woman’s business and a man should not have to worry about it (outcome)	• Attitudes towards gender norms (GEM Scale) (impact)
Community or facility	• Number/percent of vasectomy referrals (output) • Number/percent of facilities that offer vasectomy services (output) • Number of FP providers trained on male-specific FP (output) • Number of vasectomies performed (outcome)		• Number of providers trained on gender equity and sensitivity (output)
Structural	• Inclusion of vasectomy in FP guidelines/ strategies, regulations, or policies (outcome)	• Evidence of engagement of men in FP incorporated in national health standards or policies (outcome)	• Number of national level programs/ policies/advocacy campaigns that address gender equity (outcome)

The indicators can be used selectively as part of the evaluation of national programs, regional programs, and country projects. For routine monitoring purposes, we recommend that program managers and evaluators select a few relevant indicators that are important to program objectives and easy to collect and interpret. Integration of these indicators in routine health information systems is particularly important in contexts where male engagement in FP is prioritized in national FP and RH strategies. If organizations need more data, they can conduct special studies to evaluate the programs’ performance in areas of interest to staff.

We recognize that organizations adapt indicators to their specific circumstances as well as to the socioeconomic and cultural contexts in which their programs operate. This approach not only ensures that the indicators are relevant to the organization or intervention in question, but also promotes ownership of the M&E process. At the same time, we recommend that countries and organizations consider using some of the indictors listed below, as applicable.

Full indicator reference sheets for these 15 indicators may be found in the Family Planning and Reproductive Health Indicators Database. We aimed to include as much information as possible from existing indicator reference sheets, but, where necessary, we revised, added, or deleted language for accuracy and clarity. We developed new reference sheets for indicators that did not have them.

### 4. Use existing data collection approaches and methods

At the national level, the men’s survey in the DHS contains a wealth of information about men and FP. This includes: contraceptive knowledge; fertility and fertility preference; attitudes toward contraception; gender attitudes; and contraceptive use ( MacQuarrie *et al.*, 2015). However, not all country DHS include the male questionnaire. Countries with a strategic focus on and projects supporting male engagement should be encouraged to include the male questionnaire in their DHS. These data are helpful for evaluating broad trends on a longitudinal basis and for establishing program baselines. However, the data are not useful for routine monitoring, for evaluating the immediate outcomes of a specific FP project or intervention, or for gathering information from a group of men. Examples of indicators that are tracked in the DHS are:

- Percent distribution of contraceptive methods currently used by men or their sexual partners
- Percent of men who disagree that contraception is a woman’s business and a man should not have to worry about it

Indicators related to vasectomy, one of only two male-controlled modern FP methods, may be captured from routine health information records, such as the number/percent of vasectomies performed. Other quantitative facility-level indicators can be collected from facility records or service provision assessments, such as the following:

- Number/percent of vasectomy referrals
- Number/percent of facilities that offer vasectomy services

Data collection forms specific to a program or intervention should be used for quantitative indicators not covered in routine health information systems. This could pertain to facility and community-level data on service delivery, training, and outreach as well as on knowledge, attitudes and practices. Examples of such indictors are:

- Men’s condom use at last sex
- Number of providers trained on gender equity and sensitivity

Structured or in-depth interviews are a useful method for obtaining more qualitative information on knowledge, attitudes, and practices. A helpful approach is the GEM Scale, which includes 24 items to measure attitudes toward gender-equitable norms. The scale is useful for M&E of male engagement in FP because it is designed to provide information about the prevailing gender norms in a community, in addition to the effectiveness of programs that seek to influence them. Other information that can be gathered from interviews is:

- Percent of men who support the use of modern contraception for themselves or their partners
- Percent of men who share in the decision making of RH issues with their spouse or sexual partner

Last, reviews of laws, guidelines, strategies, and so forth will provide evidence at the structural or policy level. Examples of indicators obtained through such document reviews are:

- Evidence of engagement of men in FP incorporated in national health standards or policies
- Inclusion of vasectomy in FP guidelines/strategies, regulations, or policies

## Data availability

### Underlying data

Figshare: Desk Review, KIIs and Indicators for Male Engagement in FP.docx. https://doi.org/10.6084/m9.figshare.7859459.v2 ( Adamou, 2019).

This project contains the following underlying data:

- Key Informant Interviews_Male Engagement in FP.docx (de-identified key informant interview transcripts)
- Desk Review Matrix for Male Engagement in Family Planning.xlsx (list of publications identified during literature review)

### Extended data

Figshare: Desk Review, KIIs and Indicators for Male Engagement in FP.docx. https://doi.org/10.6084/m9.figshare.7859459.v2 ( Adamou, 2019).

This project contains the following extended data:

- Indicators Related to Male Engagement in FP.docx (indicators of male engagement in family planning identified in this study)

Data are available under the terms of the Creative Commons Zero "No rights reserved" data waiver (CC0 1.0 Public domain dedication).

## Footnotes

* From the IGWG “Gender Equality Continuum Tool” (2012): “Exploitative Gender Programs/Policies are programs/policies which intentionally or unintentionally reinforce or take advantage of gender inequalities and stereotypes in pursuit of project outcome, or whose approach exacerbates inequalities. This approach is harmful and can undermine the objectives of the program in the long run. Accommodating Gender Programs/Policies acknowledge but work around gender differences and inequalities to achieve project objectives. Although this approach may result in short term benefits and realization of outcomes, it does not attempt to reduce gender inequality or address the gender systems that contribute to the differences and inequalities. Transformative Gender Programming includes policies and programs that seek to transform gender relations to promote equality and achieve program objectives. This approach seeks long term outcomes by challenging the existing gender inequities and promoting positive changes in gender roles, norms, and power dynamics that drive health outcomes.”
