# Supplementary material for: Exploring gaps in monitoring and evaluation of male engagement in family planning
Source: Gates Open Res. 2019 Apr 8;3:1114. [Version 1] doi: 10.12688/gatesopenres.12927.1 (PMC6556759; doi:10.12688/gatesopenres.12927.1)
Supplement: Peer review history

# Peer review: Reviewer response for version 1

**DOI:** 10.21956/gatesopenres.14029.r27117

**Published:** 2019-06-05

**Authors:** Njoku Ola Ama

**Affiliations:** 1 Department of Statistics, University of Botswana, Gaborone, Botswana

The authors set out with objectives which have been well addressed and answered using appropriate design methods. The organisation of the work is very good and the authors demonstrated sound mastery of the English. I particularly enjoined reading the work from page to page as I did not have to struggle to understand any statement (very clear and explicit).

 The elaborate methodological approach adopted by the authors is highly desirable.

 May be the author(s) can consider using more distinguishing shading on the pie chart to allow readers who might print the work in black and white to be able to distinguish the different areas along with the key.

I have read this submission. I believe that I have an appropriate level of expertise to confirm that it is of an acceptable scientific standard.

# Peer review: Reviewer response for version 1

**DOI:** 10.21956/gatesopenres.14029.r27038

**Published:** 2019-04-30

**Authors:** Mellissa Withers

**Affiliations:** 1 Institute on Inequalities in Global Health, Keck School of Medicine, University of Southern California, Los Angeles, CA, USA

Thank you for the opportunity to review this fantastic manuscript, which outlines the authors' efforts to better understand how to measure male engagement in family planning. This is a timely and important topic, as more attention is being paid to the male partner's influence in women's family planning use. It would make a very important contribution to the literature.

 This manuscript is very well-written. It is concise, easy to follow, and well-referenced. The objectives were clearly stated.

 The introduction provides a good justification for this study, highlighting the lack of clear indicators to measure male engagement, limited data on male engagement, and complexity of measuring gender.

 The authors also make a good point that male engagement should be understood as "constructive male engagement."

 The methods were clearly detailed. It is also very useful to have the study tools and data available for review through a website for those who would like further information.

 This study is valuable in providing insight and recommendations to stakeholders (including researchers, policymakers, etc) on how to improve our measurement of male engagement. This also advances a standardized definition of male engagement in FP and a set of core indicators to use to evaluate this, which is extremely useful to those of us working in this space. 

 I had the impression that males as clients of FP is not necessarily engagement in FP as we tend to define it. Yet, most of the programs that seem to include male engagement as an objective are focusing on males as clients. Such programs might have unintended negative consequences to women. The authors did a good job on discussing the importance of a focus on male as partners and men as agents of change as being more impactful in terms of promoting gender equality.

 I found it interesting that there were no trends found in terms of programmatic approaches or goals in terms of male engagement in FP at the national levels. This highlights an important area of attention. In addition, the paper illustrates that the more widely used FP indicators (developed and standardized decades ago) rarely capture male engagement in FP. The lack of routinely collected data, as well as disaggregated data by sex and contraceptive method, are areas to explore in further research and policy. And the lack of specific, quality indicators of male engagement in FP as program objectives is clearly problematic. 

 Some small points:

 Pg 3 (Introduction) I found a placeholder for the acronym of the Interagency Gender Working Group that was blank (ie. "()").

 Pg 4 (methods) I wonder why the term "contraception" or "contraceptives" wasn't used. Also, a limitation is that all of the articles included were published in English, which was also not mentioned in the limitations section. And the KII informants were mostly based in the US.

 The online forum strategy over 4 weeks as a way to gain insight into this topic was an interesting approach.

 The only part that was a bit confusing to me was how the authors scored the indicators based on 8 standard criteria and how points were assigned. I am still not clear on that.

I have read this submission. I believe that I have an appropriate level of expertise to confirm that it is of an acceptable scientific standard.

# Response

**Published:** 2019-04-30

**Authors:** Bridgit Adamou

**Affiliations:** University of North Carolina at Chapel Hill, USA

Due to space constraints, we were not able to elaborate on the scoring criteria for our indicator review. After we compiled the indicators in the Excel spreadsheet, the study team systematically analyzed each one based on eight standard criteria, and scored each indicator based on a scale. For the binary scales, only indicators that met the criteria were assigned a point. For the criteria with a scale of one to three, indicators were assigned one point if the indicator did not meet the criteria, two points if the indicator somewhat met the criteria, and three points if the indicator met the criteria. Because of the subjectivity of the scales, three reviewers in the study team scored each indicator based on the eight criteria and an average score was calculated.

**Criteria** **Explanation** **Scale** Specific The indicator is specific to the change being measured. It is precisely formulated, not vague. 0, 1 Measurable The indicator is easily monitored, and amenable to independent validation. 0,1 Attainable The indicator requires data and information that can be collected. 0,1 Relevant The indicator is appropriate to the subject of male engagement in FP and evaluation. 0,1 Commonly used The indicator is frequently used by programs to monitor or evaluate male engagement in FP. 1, 2, 3 Validated and/or already collected in routine data collection The indicator is already validated and/or used in routine data collection, such as DHIS 2, Demographic and Health Surveys (DHS), or other validated surveys. 0, 1 Generalizable The indicator can be used across multiple types of FP interventions and is not specific to a method or process. 1, 2, 3 Applicable to FP programs sponsored by a variety of funding agencies, governments, or NGOs worldwide The indicator can be used by any program/project regardless of implementing or funding agency. 1, 2, 3
